# Patients’ experiences of internet-based Acceptance and commitment therapy for chronic pain: a qualitative study

**DOI:** 10.1186/s12891-020-03198-1

**Published:** 2020-04-06

**Authors:** Nina Bendelin, Pär Björkdahl, Mimmi Risell, Karin Zetterqvist Nelson, Björn Gerdle, Gerhard Andersson, Monica Buhrman

**Affiliations:** 1 Pain and Rehabilitation Center, and Department of Health, Medicine and Caring Sciences, Linköping University, SE-581 85 Linköping, Sweden; 2 Department of Behavioural Sciences and Learning, Linköping University, Linköping, Sweden; 3 Department of Thematic Studies/Child Studies, Linköping University, Linköping, Sweden; 4 Department of Clinical Neuroscience, Karolinska Institute, Stockholm, Sweden; 5 Department of Psychology, Uppsala University, Uppsala, Sweden

**Keywords:** ICBT, ACT, Person-based approach, Motivation, Change, Engagement, Attrition, Constructivist grounded theory

## Abstract

**Background:**

Chronic pain is a globally widespread condition with complex clusters of symptoms within a heterogeneous patient group. Internet-delivered Acceptance and Commitment Therapy (IACT) has shown promising results in the treatment of chronic pain. How IACT is experienced by patients is less well known. Qualitative studies of patients’ experiences are needed to further understand factors behind both engagement and negative effects. The aim of this study was to explore how IACT was experienced by chronic pain patients who had participated in a controlled trial.

**Methods:**

Through an open and exploratory approach this study aimed to investigate how IACT was experienced when delivered as a guided self-help program to persons with chronic pain. Eleven participants were interviewed over telephone after completing IACT.

**Results:**

Qualitative analysis based on grounded theory resulted in 2 core categories and 8 subcategories. *In treatment*: *Physical and cognitive restraints*, *Time and deadline*, *Therapist contact*, and *Self-confrontation*. *After treatment*: *Attitude to pain*, *Image of pain*, *Control or Command*, and *Acting with pain*. Individual differences as well as specific conditions of the treatment may explain variations in how the treatment was approached, experienced and what consequences it led to. Therapist guidance and deadlines for homework play complex roles in relation to autonomy and change.

**Conclusions:**

Adjusting treatment content and format based on participants’ characteristics, such as expectations, motivation and restraints, might positively affect engagement, autonomy and change. Further research on attrition and negative effects of treatment might clarify what enables chronic pain patients to benefit from IACT.

**Trial registration:**

clinicaltrials.gov (NCT01603797). Registered 22 May 2012. Retrospectively registered.

## Introduction

Chronic pain is a globally widespread condition with complex clusters of symptoms within a heterogeneous patient group [1]. Its consequences affect several domains in life [29]. Internet delivered cognitive behavioral therapy (ICBT) offers new possibilities for chronic pain treatment [23] and can diminish barriers such as geographical distances and physical limitations [4]. ICBT has shown small to moderate effects in studies on chronic pain, suggesting that ICBT can be as effective as face-to-face multimodal rehabilitation for chronic pain [11]. Knowledge of usability and patients’ experiences, expectations and motivation to internet based self-help treatments is slowly growing [3].

ACT builds on traditional CBT techniques although driven by experiential learning and focuses on different facets of psychological flexibility [31]. ACT targets the function of pain behavior within its specific context [39]. At least 13 RCT:s [40] and five systematic reviews [30, 33, 40–42] suggest that ACT can be an effective treatment for chronic pain. Also, ACT has been listed as an empirically supported treatment for chronic pain [5] and recommended as an alternative to traditional CBT [33, 42]. Low to moderate quality trials [40] have shown significant effects on pain interference, disability, catastrophizing and intensity, with effect sizes comparable to the ones in CBT trials for chronic pain [25].

Internet-delivered treatments for chronic pain may reduce pain, depression, anxiety and disability among adults [24] and children [27]. IACT specifically may effect pain acceptance, anxiety, depression, catastrophizing, pain interference, affective distress, pain intensity, pain disability, and fear-avoidance, with effect sizes varying from small to large [45]. However, IACT remains a relatively under-researched area. For example, the use of multimedia has been found feasible and appreciated when delivered with therapeutic guidance [12] although less usable without guidance [22]. Even though multimedia may facilitate experiential learning in IACT, it has not been shown that it improves outcome, completion or satisfaction.

Patient attrition in internet interventions is another under-researched area [11, 36]. As the risks of attrition might be difficult to discover prospectively [35], more knowledge is needed about the reasons behind attrition and its negative effects [4]. Wider research have shown that self-discovery, acceptance and self-efficacy may enable self-management, meanwhile distress and declined motivation have been described as hindrances [21].

Thirdly, how to deliver guidance [3] and the role of the therapist have been discussed [20]. Patients report that therapist guidance may enhance engagement and self-management [7, 21, 22]. Also, results are often better in guided ICBT compared to un-guided [6]. Individualized guidance might help patients gain from pivotal interventions [7]. However, ICBT for chronic pain has so far been successfully delivered without individual adjustments [11, 19]. Tailored treatment is relevant when there is a variation in symptoms or consequences, or when pain is one of comorbid syndromes [4]. Adjusting after learning needs might balance cognitive demands while covering all relevant interventions in IACT [9, 43]. Although it is still unclear whether tailoring might improve engagement or outcome [38], a growing knowledge suggests that adjusted treatment might be necessary for self-managing pain over time [21].

Qualitative evaluations of IACT for chronic pain have found that goal-setting may be an important intervention for ongoing practice of self-management skills [7] and can serve as a key element to support behavior change [22]. Interview studies [7] and research on the e-therapist’s role [22] have been suggested as important for further research as therapist guidance may play a central role in engaging participants. The person-based approach has evolved as a qualitative approach that aids the development of internet-delivered interventions [37, 48]. It may be applied at different stages of development, for example to identify user reactions, needs and challenges when evaluating acceptability and feasibility [46]. One purpose is to optimize engagement and usability in future designs, with awareness of the experiences of the users [46].

### Aim and research question

The aim of this study was to explore how IACT was experienced by chronic pain patients who had participated in a controlled trial [12].

## Method

Since chronic pain patients’ experiences of IACT is a novel area of research, a grounded theory-based method for analysis was chosen combined with a constructivist approach [18]. In a novel stage of intervention development, a qualitative approach may be useful to openly explore the area, by describing and seeking understanding in participants’ experiences [14]. The person-based approach guides an intervention developer to both understand how patients perceive and engage in an intervention, and apply results to enhance feasibility and acceptance [46]. Its user-perspective is unlikely to present the experiences of the entire population. Rather, it may highlight complexity, variations and changeableness [44]. As the in-depth approach is a key element [46], in-depth semi-structured interviews were used.

### Participants

Participants in the present study had completed an IACT for chronic pain [12]. The treatment program was based on ACT principles [31] and consisted of seven chapters of different themes, for example Alternative to control, Willingness, Thoughts and Emotions and Life values. Each chapter consisted of a written part and audio tracks with metaphors and present moment exercises. All chapters also included a number of exercises for the participants to do by themselves. Participants had weekly therapist contact via the internet and also telephone contact at two occasions during the treatment. Results of the trial [12] showed improvements in favor for the treatment group on pain acceptance, Chronic Pain Acceptance Questionnaire, (*d* = 0.41); subscale pain willingness, (*d* = 0.49); subscale activity engagement, (*d* = 0.60), Hospital Anxiety and Depression Scale anxiety, (*d* = 0.18); depression, (*d* = 0.44), two subscales from Coping Strategies Questionnaire (CSQ); catastrophizing (*d* = 0.51); praying/hoping (*d* = 0.28), and two subscales from Multidimensional Pain Inventory (MPI); pain interference (*d* = 0.56); affective distress (*d* = 0.30). Results were maintained at 6 months follow up, except for the MPI-Pain interference subscale which was improved (*d* = 0.32). Significant effects were not found on Quality of Life Inventory, The Pain and Impairment Relationship Scale, the subscales diverting attention, reinterpret pain sensations, coping self-statements, ignore pain sensations, increasing activity level and pain behaviours from CSQ or on the subscales pain severity, life control, support, punishing responses, solicitous responses and distracting responses from MPI. Additional data is presented in [12].

Of the 76 patients who participated in the IACT program for chronic pain, 18 completed all modules. To collect patients’ experiences of the treatment program as a whole, a selection was made, so that only those 13, who had completed the entire program within the designated treatment time, were asked to participate. As one person was excluded due to health reasons, the remaining 12 were asked to participate. Of these, 11 accepted; 8 women and 3 men with mean age 46 years old (spanning from 27 to 86) (1 declined for an unknown reason). Pain duration varied from 2.5 to 22 years. A wide range of pain diagnoses were represented, and they all had chronic pain in at least two localizations. All participants received compensation from the social insurance agency. Four participants worked part-time or studied. An equal amount had high school education as had college education.

### Data collection

Nine months after treatment, participants were interviewed over the telephone, by one of two interviewers (second and third author). Interviews started with background questions about employment status, pain demographics and duration and their health status (see Additional file 1). The following questions throughout the interview were open and explorative in a semi-structured way, in order to produce a free story of their experiences [47], for example “Tell me about the treatment you’ve received”. Deepening and clarifying questions were used to help participant formulate their experiences. The interview guide was revised with minor changes after an initial pilot interview. Mean length of interviews were 43.7 min (spanning from 33 to 57). Besides transcripts of interviews, the interviewers’ impressions were also written down for later inclusion in the analyses. The two interviewers listened to each other’s interviews and completed each other’s transcripts. A supervising researcher (fourth author) read parts of the material and contributed with ideas to the analysis.

### Analysis

**Fig. 1:**
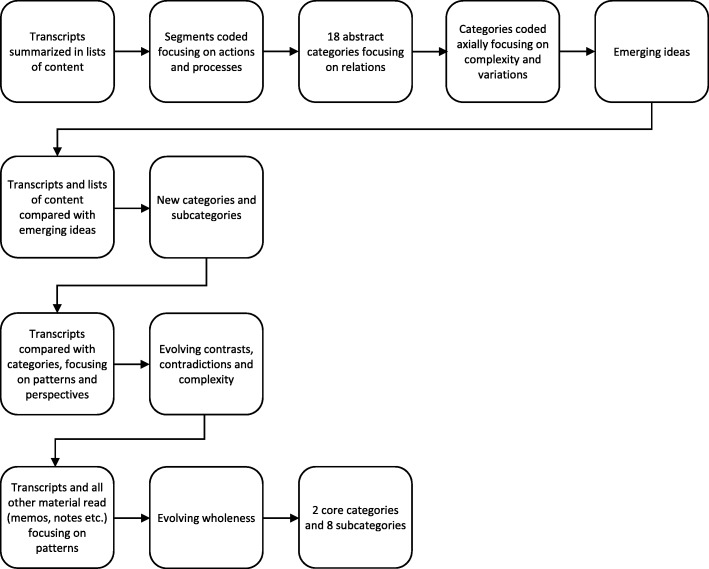
Flowchart of process of analysis

At first, all transcripts were read through repeatedly for the interviewers to get familiar with the material and get an overview. Each transcript was summarized in a list of content (see Fig. 1). All material (transcripts and lists of content) was coded openly segment by segment. The material was coded while moving quickly through it, in line with grounded theory [16]. Codes were simple, focusing on actions and processes in participants’ narratives, as recommended in constructivist method of analysis [17]. Transcripts were read and coded separately but simultaneously by both interviewers and continuously discussed. As codes emerged, they were joint in 18 more abstract categories.

Relations within and between categories were discovered as they were coded axially. Emerging ideas and hypothesis were asked to the material, by constant comparative analysis [15], in order to elaborate its complexity and variations. This resulted in a wholeness with primarily new categories and subcategories. Transcripts were read again to be viewed from the eyes of new ideas, in attempt to find new patterns or perspectives. In doing so, categories were continuously compared with initial data. In the latter part of the analysis process, further contrasts and contradictions were brought to light and a complexity within each category emerged. All material was once again read through so that nothing substantial had been lost in the wholeness. Meanwhile, clarifying quotes were noted. In the final stage of analysis, patterns were sought in attempt to understand why participants experienced the program differently, learnt diverse things as well as changed in disparate ways. A meaningful wholeness was expressed in two core categories and 8 subcategories.

In line with constructivist grounded theory [15, 17], pre-knowledge of the two interviewers were written on memos and were taken account for in discussions of preliminary ideas early in the analysis process. In collaboration with a supervisor, the influence from pre-knowledge was elaborated to enable interactive movement from data material to theoretical knowledge. In order to keep an open mind while collecting and analyzing data, comparisons with previous findings were primarily done in the latter part of the analysis process.

Quotes in Table 1 and in written text are presented to illustrate and clarify category content. Quotes are grammatically corrected and words have sometimes been replaced to ensure anonymity.

**Table 1:** Overview of core categories, subcategories and examples of quotes from participants (P)

Categories	Examples of quotes
IN TREATMENT	
Physical and cognitive restraints	*For my part, it was tough, maybe partly because of physical hindrance, since my hands and arms and knees and everything hurts, which makes it difficult to work on a computer. That’s why typing those assignments and all was so hard. Also, my knees start aching after sitting on a chair for some time. So, I suppose it was actual physical obstacles that made it hard* (P 1)*.*
	*Well, you know, getting your head around “what is this and what does it really mean?”. Because, I mean, being in pain, you are a bit dull and sometimes you can’t figure things out. So, one had to stay really focused, that’s what I felt* (P 2).
	*What I had to do was to ask my best friend to come over and she would read it out loud for me while I was lying down resting, and also helped me write. Then I tried on my own and some stuff you must think about yourself and all that. She actually helped me write some stuff because I was under pressure getting it all done*. (P 3).
Deadlines and time	*Say you’re going once a week to see a pain therapist or something, you work stuff up to talk about when you get there. But maybe you’re not in the mood at that exact hour. Here, you can do it as it comes, so to say and that’s another way* (P 4).
	*And maybe some need a shorter period with more frequent contact. When doing this, perhaps some flexibility would be good, to let the patient choose really, some parts (…*) *It’s important to listen to that person, how he or she works and such, because then treatment can be adjusted in the best way, you know* (P 5).
	*I mean when I come home, I have to rest first, then you know, it takes time for me. So, an hour or so goes by (…*) *And one hour every evening means less time for resting. Then I need to reflect and give it some thoughts and work some more on the computer and ask someone to come over and write for me. So, several hours went by* (P 3).
Therapist contact	*She said it’s common that pain gets worse under pressure. It doesn’t matter where or how it hurts. Stress makes it worse. Good to know really, that you’re not nuts.* (P 6).
	*When I was thinking, I’m not getting this, and what does it really mean? Even if my writing was confusing, we could go through it. It felt good when they called to follow it up to make sure it was carried out, and to get answers to my questions and such* (P 2).
	*But that was actually the benefit because when she questioned me back and so, I had to think about pain in other ways. To not let it tear me down, rather put it aside, let it be, try not to focus on it, do something else, do something fun (…*) *That’s what you need, a push, some back-up to get started when you’re in pain all the time. Being stuck in pain makes you narrow-minded. That’s why the communication* via *computer and telephone was truly important to me.* (P 7)
	*It’s easy to get insecure. Or, often I just need to hear another voice saying “How are you? How’s it going?” (…*) *Not only sit there with a text (…*) *A bit lonely.* (P 8)
Self-confrontation	*Well, it’s been so long you tend to forget how it was and who you were. As for now, I’m mostly a pain patient. It’s a good thing to get a feeling of and remembering how I was before and that I do still exist even if I’m another person or something. I’ve still got my … not merely pain, but you know, I’m myself.* (P 3).
	*Well, I suppose it was demanding in the way that … Everything psychological is demanding really; when you’re putting things into words, formulating goals, really feeling, describing and explaining a lot of different things.* (P 9)
	*It was in my head throughout the day the entire treatment time, I felt. Things I ought to do, think over and certain assignments for specific situations. Actually, quite demanding emotionally.* (P 9)
	*It mustn’t go too fast. It takes time to be willing to accept. Accepting that you’re never going to be well and realizing that things are the way they are. It’s awfully hard really. You just can’t.* (P 5)
	*“I’m allowed to focus on myself, to think and contemplate over my situation”. (P 3)*
AFTER TREATMENT	
Attitudes to pain	*Because of the treatment, there’s a continuity all the time. Every week, answering those questions, it actually became a reality (…*) *It helped me accept this. To actually see this is what it is. To me that’s the greatest change. I probably consider my pain more often now, accepting it kind of. Now, this is it. It takes away like a quarter of me and this is the way it is now.* (P 4).
	*A new attitude to life, I’d say. Stop thinking of what’s coming next. Rather, do it now and do what you can. There’s no idea waiting for better times, because that might never happen since it might never get better than this. And that thing about focusing on the here and now, and not think about other stuff. As when my son comes home from school telling me about his day – I really shouldn’t plan dinner in my head meanwhile. Rather actually listen to him. That I learned during this treatment.* (P 1)
	*But you know, it’s really about facing up to the situation and do the best possible thing. You don’t have much choice. If you don’t try, you’re lost.* (P 2)
	*Imagine all those people running through life without experiencing the here and now* (P 1).
	*To be willing to have. To have what I have so to say. I think I had some trouble relating to that since I’m still trying to find out what this is and I’m still looking for treatments that could ease pain* (P 9).
	*I forced myself because I really wanted to, you know. I wanted a change in life so badly.* (P 4)
	*It costs nothing to try. It can’t really get any worse.* (P 6).
Image of pain	*I had a grey lump. It was my pain and I could place it on the table and look at it (…*) *When blaming that thing instead of myself or something else, I can focus on that grey lump which I’d like to beat to death. To get rid of pain (…*) *I mean, really, the only thing you want is to run away from it. All you want is to get away from it. Far, far away. Think of something else. But like this, it was almost as if I was personalizing pain. Because I made it into a thing, something concrete, as a grey substance.* (P 7)
	*It feels good to sometimes be able to take that substance and move away from it a bit. It doesn’t have to be far. But simply being able to get it off me, hoping it stays put and doesn’t come after.* (P 7)
	*That guy in my body, who is running about hurting me (P7)*
	*Now I sort of feel I’m not carrying pain in a bag, I’m rather holding on to a thin, thin fabric that flows around me (…*) *Instead of carrying that bag which is damn heavy and hurting my hand, I carry a thin, thin fabric that flies with me.* (P 4)
	*I’m in my pain in a different way now. I can’t see it in front of me. I’m not trying to keep it at a distance. Rather I feel I’m with it most of the time.* (P 9).
Control or command	*It’s like I’m in command after all. If I’ve set my mind on doing this thing today, pain won’t keep me at home. It means the world to me, that my disease doesn’t knock me out, rather I can knock it out a bit as well.* (P 2).
	*As I understood it, you’re not supposed to be focused on controlling pain all the time, but for me controlling pain makes me able to do more. When I do too much it might result in not being able to do anything for three weeks.* (P 1).
	*I do understand it logically (accepting pain), but I can’t help myself from trying to overcome my pain in other ways, by trying to get help in other ways. Since there are so many other methods.* (P 9).
	*I suppose relaxation techniques helped me the most so to say (…*) *I did … I have one that’s fairly good. I suppose that’s the one I listen to even now, maybe once, twice a month. Well it does help sometimes if I turn off the TV and just listen to that tape and take it easy for ten minutes. (P 6)*
Acting with pain	*Pain’s not important. It might be the thing that affects my life the most, but it’s still not important … it’s much better for me to simply do those things I value … It might lead to me being in bed for five days afterwards. But I did do something that mattered to me (…*) *So constantly, I pay in pain … But on the other hand, I did really achieve something, because if you’re committed to pay with five days of pain for half an hours’ work, then you really know what’s important in life, you know.* (P 5)
	*Sometimes on a Saturday or Sunday, something is going on and I want to join. And then the consequence might be that I’ll be lying down Monday and Tuesday instead. Yeah, that’s tricky. Constantly one needs to prioritize. Choose what not to do (…*) *It’s extremely difficult to get away from it, because it’s always about being responsible, responsible, responsible. And of course, I want to! I do want to be a responsible person. But you simply can’t when you’re in pain.* (P 5)
	*I want to feel I can do something. I can’t just stay at home. Because there are also good times, actually. Even If I’m never getting well altogether, I do have good times. And why shouldn’t I be able to do something useful of them?* (P 2)
	*Actually, that I’m doing something of my time when I’m well enough to be active … I suppose that’s the greatest change as I see it.* (P 7).
	*It gave me some perspective; I’m not totally stuck with “I can’t”.* (P 8).
	*Since I can’t ride anymore, because of pain in my knees and all, it’s like, just because I can’t ride, I shouldn’t spend time with horses at all. But now I see that, well, its’s all right to go there and simply cuddle for a while or something. I don’t know really, but maybe, being satisfied with that small part at least. Instead of “Well if I can’t have it all I don’t want any of it”- rather I better go out and do something tiny then not doing anything at all. Which makes me feel better as well*. (P 1).

## Results

**Fig. 2:**
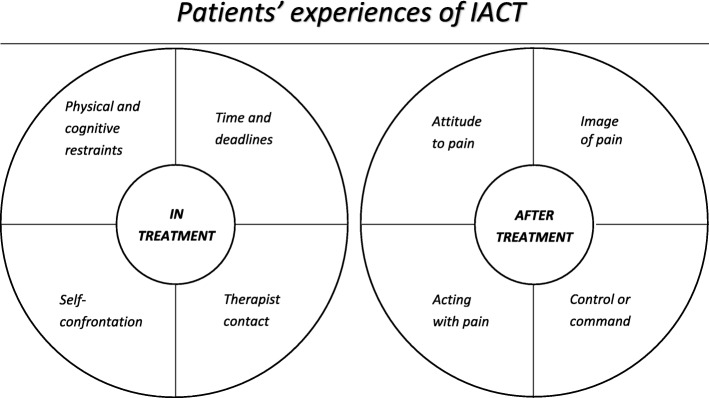
Results presented in core categories and subcategories

Results are presented in two core categories: (1) In treatment and (2) After treatment. In treatment covers participants’ experiences during treatment, consisting of subcategories Physical and cognitive restraints, Deadlines and time, Therapist contact and Self-confrontation. After treatment covers participants’ experiences after treatment, and consists of subcategories Attitudes to pain, Image of pain, Control or command and Acting with pain (See Fig. 2).

### In treatment

The interaction between participants’ characteristics (as physical ability, expectations, motivation and knowledge) and the specific treatment (IACT) affected their experiences. These are described in the following subcategories and illustrated by quotes in Table 1.

#### Physical and cognitive restraints

Muscle and joint pain made physical assignments as exercising difficult. Strained neck affected computer-based assignments, as did pain in fingers, hands and arms. However, computer-time was also seen as an opportunity to focus on sitting posture. Attentional difficulties, poor study technique and loss of energy, made theoretical parts harder to comprehend, especially sustaining focus while processing written information and expressing thoughts and feelings. Reading printed text lying down was perceived as helpful, as was paced reading, although the interruptions were a nuisance. Participants wished for all material to be in audio format. Support from relatives and friends, repeated resting and acceptance skills were helpful when doing assignments. For some, determination and deadlines eased commitment.

#### Deadlines and time

Participants appreciated the opportunity to choose when to do assignments, for example at night or when in the right mood. Generally, participants suggested that individually adjusted deadlines would reduce stress, allow pacing and deep learning, and enhance engagement. Treatment was perceived as stressful and handled by putting other commitments on hold, prioritizing certain parts or integrating assignments in everyday tasks. Some found time disposition difficult. Some felt monitored, obligated and pressured as to pass an exam. Others appreciated planning their own time and someone described treatment structure as essential to complete.

#### Therapist contact

Emotional distress and technical problems were overcome by help from the e-therapists, described as mentors, contact persons or “the woman in charge” (P10). Therapists were perceived as empathetic, understanding, experts, educated, supporting and confronting. A few participants did not remember the contact or attribute it any significance, meanwhile others perceived it as a necessity to complete. Some felt lonely and longed for additional, verbal or live contact, for example when doing assignments. Some felt liberated by focusing on treatment content rather than “*performing at sessions*” (P4) or “*trying to look cheerful and happy*” (P2). Contacting the therapist if necessary was possible, perceived as luxurious, however rarely done. Participants’ characteristics, as earlier experiences from health care were mentioned as they described their needs and wishes for contact.

#### Self-confrontation

Participants were exposed to feelings, thoughts and memories while reviewing their life, previous persona and self-image. This led to mourning, self-pity and a sense of personal integrity. Shredded life ideals were grieved while setting goals, resulting in sadness, hope and determination. One participant described that mourning the past that never happened and facing the boundaries that come with pain “*eventually leads to focusing on what is doable*” (P1). Writing about feelings and thoughts exposed participants repeatedly to themselves, which was perceived as important and necessary yet intense, tough and demanding. Committing to treatment was essential for self-exposure work resulting in existential reflections, increased understanding and acceptance of self. “*Getting close to oneself*” led to self-respect and calmness, which for some was the most significant and essential experience of the treatment. However, others perceived self-exposure as too demanding, time-consuming or hard. Lack of interpersonal exchange in this phase was both appreciated and seen as a deficit.

### After treatment

Impact of treatment induced changes varied. Individual characteristics influenced participants’ focus in treatment. Experienced consequences after treatment are described in the following subcategories and illustrated by quotes in Table 1.

#### Attitudes to pain

Treatment helped participants see pain as a part of life. Experiential exercises, for example on life values, influenced their view of life, inspired to accomplishments and gave insights that were generally applied. Present moment exercises led to both practical every-day changes and a new approach to life. Acknowledging that pain would not be cured helped participants be present and do things that mattered. Phrases as “*this is my plate*” (P8) were used to describe a life including both pain and things they valued. Participants felt privileged, having experienced something rare that “*everyone ought to try*” (P5). They showed a new view of life and saw themselves as more accepting than others. Some participants described both an explicitly changed attitude and a strong desire to change their way of living before and during treatment. Others were searching for new coping techniques, appreciating specific mindfulness exercises, however approaching treatment more passively, without experiencing a shift in attitude. One person found it impossible to reconcile with a life in pain, although theoretically appreciating the concept of acceptance.

#### Image of pain

Modifying the mental representation of pain by visualization exercises changed some participants’ view of pain, reduced self-blame and alienated self from pain. Distancing from pain helped participants become more attentive to own wishes rather than acting upon pain. Visualizing pain as one object among others, taking some but not all space, metaphorically speaking on a table or in a rug-sack, helped participant look forward while being in pain. Disengaging from pain and picturing pain as an object outside of the body helped participants not to feel dependent or controlled by pain. This mind work demanded effort, imagination, skills training and reconciliation that pain was a constant companion in life, as “Laurel and Hardy”. As preparation before activities and as a mean to act consciously meanwhile, visualization brought a sense of freedom. Modifying the vision of pain made life easier for some, although others did not experience a modified perception of pain at all.

#### Control or command

Repeated practicing of mindfulness helped participants tolerate pain, stop it from escalating, find peace in mind and reduce stress. Such skill training both aided with managing pain in the here and now and evolved into new attitudes to pain. For some, the most essential gain was the ability to move away from fear and panic, to a state of calmness and control. Others learned to not be held back by pain or to stay in charge themselves, rather than calling for help. New relationships to pain emerged, for example described as “*to disconnect what hurts*” (P11). The same participant also stated, “*set one’s mind on something else for a while*” (P11) and “*not to care about it*” (P11). The intensity of pain could be lessened by dreaming of the future or imagining a pleasant site. Mindfulness was repeatedly mentioned as a mean to take command over pain and prevent it from taking over. The new attitudes helped them cope and adjust activity level. Instead of forcing themselves or clenching their teeth to distract from pain, they slowed down and felt pain. Opening to and focusing on the surroundings helped with handling one thing at a time when challenges occurred.

Although all participants developed new skills, some applied these as tools to control pain. Some feared that catastrophic consequences would come from letting go of pain control strategies from years back and would not take the risk. Others perceived accepting pain as too tough, and yet others were still searching for ways to get rid of pain, for example by practicing mindfulness to relax or refine their breathing techniques.

#### Acting with pain

Participants reported new ways to interact at work, with friends and family, in their spare time and in their everyday life. They mastered their lives, were able to do what was meaningful despite pain although sometimes paying a high price, for example handling other’s reactions to their choices. One person said she lived the life she values and did not find pain debilitating.

All participants wished to be active. Facing up to pain, seeing it as part of life and staying present while working, helped with communicating at work and adjusting work performance with pain in mind. However, some realized they needed to stop “*running away from pain*” (P2) by overdoing activities on good days to prove their ability. Some were overwhelmed by pain and although knowing what was necessary to improve their health, they did not take the leap from thought to action.

Participants talked about making the most out of good times and accepting bad times. They noticed nuances in pain and saw it as something else than a constant follower. Someone started to say no to friends when there was no energy left. Someone else agreed to do things that mattered, knowing it would have consequences. Rather than thinking either or, they focused on what they could do. Some participants described this new flexible attitude as their most essential change. They stopped waiting for pain to go away or for their abilities to improve. Instead of excluding activities, engagements and commitments, they approached them differently and saw them as opportunities rather than demands to perform. This new way of acting brought along new consequences, as dealing with others’ reactions when staying true to one’s conviction, holding back when impulses were strong and keeping up the plan when pain got worse. Participants also experienced that changing patterns of behavior also meant falling back sometimes. Courage and abilities such as flexibility and staying present, are described as important when committing to meaningful activities and adjusting behavior, meanwhile being aware of pain. Expressions in line with “*doing something meaningful when I’m able to*” (P1) are common for several participants and seems to be a general new approach after treatment.

## Discussion

The aim of this study was to explore how IACT was experienced by chronic pain patients. The results show a substantial variation in participants’ experiences of how treatment was carried through, what consequences it led to and its importance. Treatment helped participants take command over pain in a life-changing way and increase their valued activities. However, some merely added a mindfulness exercise to their previous coping skills and otherwise lived on as before. Likewise, some shifted into a more willing and present approach to pain, meanwhile others showed interest for this new way but chose to continue as before. On this spectrum some described treatment as life evolving meanwhile others appreciated a single aspect of treatment. Individual differences and the specific conditions of the treatment might explain the differences.

### Autonomy

There are variations in how patients work in ICBT [8] and how they motivate themselves [7, 13]. Adapting to treatment format goes quicker for those who easily work structured and independently [8]. This was seen in those participants who found treatment important and life evolving. Other participants completed treatment but without experiencing autonomy in working with assignments nor describing essential gains. Deadlines for treatment assignments and therapeutic alliance motivated some to carry through in treatment, as did commitment to the study and avoidance of feeling guilt or failure. For some, deadlines brought on a structure that promoted autonomy which has been seen in another patient group in ICBT [9]. For some, deadlines were found stressful, also previously seen [34] and led to negative emotions of guilt, nevertheless motivating them to complete. Deadlines gave a structure that compensated for cognitive difficulties for some. Others found deadlines too stressful and therefore excluded certain parts. Stress and limited problem-solving ability have been described as barriers for self-management [21]. Stretched treatment time has been found to prevent attrition amongst youths with chronic pain [28]. A request for a stretched deadline might be a signal of attrition [35]. Although extra time to finish an assignment might be helpful for some, it risks disrupting treatment process and structure.

### Behavior change

All participants mentioned changes they had made in their lives during treatment. Treatment induced behavior changes that effected life in a positive way was reported in another qualitative evaluation of IACT [22]. As in the present study, the degree of behavior changes over time varied among participants. In wider research, variations like these have been suggested to depend on the extent to which treatment was applied in participants’ lives [8]. Some participants were actively seeking new ways of living and also found new ways of acting with pain and made longstanding changes. Others fell back in old habits, held on to old strategies or struggled to let go of these, meanwhile telling themselves to apply new skills. There were also participants who decided not to change the way they lived or dealt with pain. On this spectrum of behavior change, some participants found a new attitude to life and others became stagnated in previous habits, which has been observed before [10]. Participants described that their changed attitude to pain were affected by the process of confronting thoughts, feelings and pain. They mentioned calmness and self-respect as results from this process. For some, this was the most essential part of treatment. Self-discovery has been described as an enabler in self-managing pain [21]. However, some participants found the self-confrontation part too demanding and too hard to do by themselves. If negative effects would be reported during ICBT [4], additional therapist contact could be provided to guide the self-discovery process.

### Psychological flexibility

The ability to balance activity and rest leads to a sense of control in the everyday life of pain patients [32]. The present participants described that they prior to treatment had either given up on activities or acted without consideration. Finding a balance between fighting with pain and giving in for pain seemed to have helped participants to move away from those extreme positions. Pacing activity level by using acceptance strategies has been observed in IACT [22]. Likewise, participants in this study, in various degree showed a new mindset of flexibility towards acting with pain. Those participants who used to push themselves, described how they after treatment sometimes choose to stay home and rest. Some kept their prior activity level but choose more carefully what to engage in after treatment. A general new behavior among participants was to pause and be sensitive to ones’ needs before choosing what to do. This is a token of the psychological flexibility that ACT aims at [31]. Acceptance, self-efficacy and self-discovery has been described as drivers for optimal self-management [21]. Especially self-discovery has been suggested to elicit change [21]. In the present study, some participants were set on discovering new perspectives on pain and themselves and subsequently experienced the treatment as life-evolving. Meanwhile others were looking for new coping strategies, finding the self-confrontation part too demanding and hence did not experience change in the same degree.

### Treatment expectations

One reason for the discrepancy between participants’ changes in treatment might be their expectations. Participants who stated that they had gained a new approach to life, also described that they prior to treatment was set on such a change. Others described that they wanted something new to help with pain. Being willing to make behavior changes in the presence of pain might be difficult alongside seeking new methods to reduce pain.

Since pain management sometimes focus on controlling pain and reducing symptoms, ACT may differ from patients’ previous experiences. This may cause confusion and alienate patients from the treatment rational. It has been suggested that therapeutic guidance might be necessary to help patients engage in IACT [22]. It has also been suggested that patients with chronic conditions as pain, with comorbid depression and social withdrawal, are less likely to engage in a new treatment approaches, due to previous unsatisfied treatment attempts [35]. The present treatment contained weekly homework assignments which encouraged participants to apply what they had learnt and practice new skills. Nevertheless, there are vast variations in participants’ reports of changed behavior. An active approach to ICBT has been found to elicit change [8] and committing to long-term goals have been suggested to foster continuous self-management skills [7]. In the light of the present results, it might be beneficial to explore participants’ perceptions prior to treatment to enable an active approach to IACT, perhaps through additional guidance.

### Therapist guidance

Participants’ experiences of therapist support differed widely. Some described themselves as “the primary therapist” and did not wish for more support. Some participants appreciated the absence of a therapist since working on their own meant the success was a result of their own doing. Furthermore, some described that they were more honest when writing, since they did not need to handle another persons’ reaction to their most sincere feelings, thoughts and dreams. Guidance was perceived as helpful while reviewing previous coping skills and recurrent behavior patterns as well as when stating life values. Some participants appreciated the guidance although they longed for more, for example while exploring pain-related thoughts and feelings. Some longed for other contact.

Participants have previously mentioned that additional therapist guidance could improve their engagement [7, 34]. This has however to date not been shown to be more effective than regular therapist contact in ICBT [20]. It is yet to find out whether therapist guidance on demand could raise completion or satisfaction, if not outcome. Nor is it clear how it would affect participants’ perception of autonomy or self-efficacy. Patients with chronic illnesses, especially pain and those with long duration of symptoms are at risk for dropping out of internet delivered treatments [35, 36]. Their motivation to engage are recommended to focus upon, especially the benefits of internet delivered interventions for their specific needs [35]. Also, shared decision-making and person-centered communication has been described as important for fostering self-management skills [21]. Participants in the present study had vast and sometimes opponent perceptions of therapist contact. Considering this, one future question might be weather directed therapist guidance, focusing on goal-setting and self-exploration, could help chronic pain participants to engage in and prosper from ICBT.

### Limitations

To collect experiences of all parts and the length, pace and effort of the treatment, only those who completed the IACT pain program were selected. Even though a sample size of eleven is comparable to the ones in similar studies [21, 22], it reduces generalizability. However, at this stage of development generalizability may be low [26], as the purpose is primarily to collect experiences for further development [47].

To generate ideas on what ease as well as obstruct participation in IACT, it is essential to also include persons who did not complete treatment, who are critical or experienced negative effects. A few participants described themselves as “good students” in relation to the treatment, which has been previously described as problematic [2]. Also, participants may have held back on criticism, if interviewers were associated with the treatment itself. However, these interviewers had limited experience in IACT, which might have brought an open-mind to previously neglected opinions, as negative experiences.

Interviewing retrospectively makes it possible for participants to describe long-term consequences of treatment. However, influenced or loss of memories are to be expected. Even though telephone interviews cannot grasp non-verbal communication, these participants’ accounts were evaluated as open, personal and at times very rich in details. Also, follow-up questions in the transcripts together with interviewers’ notes show that the interviews were reflecting and elaborating, despite the distance in time and location.

The analysis was repeatedly grounded in the original data throughout the analysis process (see Fig. 2), however not in additional data. It is motivated to ground an analysis in only the primary material at a novel state of research [44]. The analysis could be considered saturated given the many contrasts, contradictions and complexities it resulted in.

## Conclusions

Considering the variations in patients’ experiences and perceived changes, it might be plausible to adjust treatment format to suit the different needs of chronic pain patients. It might also be feasible to direct therapist guidance to the processes of goal setting and exploring pain-related thoughts and feelings. Finally, this study suggests that individual expectations and restraints might point to risks of attrition and in the long run pose as barriers to self-managing pain.

We do not know if adjusting treatment format based on pain patients’ needs would render higher engagement. However, we suggest that providing easy-to-read texts and multimedia may enable patients to choose a format that meet their requests and needs, hence reducing barriers for self-management. Since little is yet known on how to reengage a patient in risk of attrition, we suggest further exploration of chronic pain patients’ expectations, motivation and needs, prior and during IACT.

## Supplementary information

**Additional file 1.**

## Data Availability

The data analyzed during the current study are not publicly available due to the anonymity of the participants but are available from the corresponding author on reasonable request.

## Abbreviations

ACT: Acceptance and Commitment Therapy
CBT: Cognitive Behavioral Therapy
ICBT: Internet-delivered cognitive behavioral therapy
IACT: Internet-delivered Acceptance and Commitment Therapy
