# Supplementary material for: Patients’ experiences of internet-based Acceptance and commitment therapy for chronic pain: a qualitative study
Source: BMC Musculoskelet Disord. 2020 Apr 6;21:212. doi: 10.1186/s12891-020-03198-1 (PMC7137329; doi:10.1186/s12891-020-03198-1)
Supplement: Supplementary file 1 — Additional file 1. [file 12891_2020_3198_MOESM1_ESM.docx]

Interview guide (semi-structured),

Open questions and *examples of follow-up questions*.

**Introductory questions pre recording**

Occupation status:

- Please tell me about your current situation concerning work and employment.

Pain demographics and duration:

- Could you describe your pain please, so that I can get an idea of how it’s like for you?

Health changes post treatment:

- How has your pain changed since this last summer? *In what way?*

**The treatment**

Description of the intervention, first impression:

- Tell me about the treatment you received? *How would you describe it? What was your first impression like?*

Treatment activities:

- Tell me about what you did during treatment.

Explicit memories, prominent parts, concepts and exercises:

- What do you remember from treatment?

*Tell me about your most prominent memories from treatment?*

*Could you describe something from the treatment that was essential to you?*

*Did some of the metaphors, words or concepts make an impression on you? How?*

*Did some of the practical exercises make an impression on you? How?*

Difficulties, hindrances for completion, comprehensive parts:

- Were there any difficult parts of treatment, to you?

*What was the most difficult part?*

*What obstacles, if any, made it difficult for you to go through treatment?*

*Were there some parts of treatment that you experienced as easier (to grasp or to practice)?*

Therapist contact, experiences:

- Tell me about the contact with your e-therapist? *How did you communicate? How did you experience that?*

Experiences of internet-delivered treatment:

- How was it like for you to be in treatment via the Internet?

Prior expectations:

- What were your expectations like before you started treatment?

Changed perception to treatment meanwhile

- Did your attitude to the treatment change in any way meanwhile? *What changed and how?*

**Change**

Treatment effect

- Was the treatment helpful to you? *In what way?*

Changes, positive and negative:

- Did anything change in your life, due to treatment? What changed? *Which positive changes? Which negative changes?*

Practical changes in everyday-life:

- Did the treatment result in any practical changes in your every-day life? *In what way?*

Perception of pain:

- Would you care to tell me about how you perceive your pain?

*Would you say that you perceive your pain any different after treatment compared to prior to treatment? In what way?*

Coping:

- How do you cope with pain? *Are there differences compared to how you coped with your pain prior to treatment?*

Reasons for change or non-change:

- In your opinion, what contributed to these changes?
- Alternatively: From your view, what reasons might there be to why change didn’t occur for you?

**Retrospective thoughts**

Most important lesson/insight:

- Looking back today on the treatment you went through, what’s the most important thing you learned?

If you would do it once over:

- Are there things you would do differently if you were to do it again?

Suggestions on content or format:

- If you could, what would you alter concerning content and format of the program?

Feelings towards treatment

- What do you usually feel when you think of the treatment?

**Closure**

New thoughts after this interview:

- During this interview, did anything new come to your mind concerning treatment that you haven’t thought of before?

Anything else I should know:

- Is there something else you think I should know to better understand your experiences of the treatment?

**Post recording**

Experiences of being interviewed:

- How was it like for you, being interviewed?

Questions to the interviewer:

- Do you have any questions for me?

Thank you!
